# Plant-Assisted Synthesis, Phytochemical Profiling, and Bioactivity Evaluation of Copper Nanoparticles Derived from *Tordylium trachycarpum* (Apiaceae)

**DOI:** 10.3390/biom15121693

**Published:** 2025-12-04

**Authors:** Venos Saeed Abdullah, Kamaran Younis M. Amin, Hawraz Ibrahim M. Amin

**Affiliations:** 1 Physiotherapy Department, Erbil Technical Health and Medical College, Erbil Polytechnic University, Erbil 44001, Kurdistan Region, Iraq; venos.saeed@epu.edu.iq; 2 Department of Chemistry, College of Education, Salahaddin University-Erbil, Erbil 44001, Kurdistan Region, Iraq; kamaran.younis@su.edu.krd; 3 Department of Chemistry, College of Science, Salahaddin University-Erbil, Erbil 44001, Kurdistan Region, Iraq; 4 Department of Chemistry, Pavia University, 27100 Pavia, Italy

**Keywords:** *Trodylium trachycarpum*, copper nanoparticles, plant-assisted synthesis, antimicrobial, antioxidant, enzyme inhibition, drug discovery

## Abstract

*Tordylium trachycarpum* Boiss. (Apiaceae) has long been used by traditional healers in the Kurdistan Region of Iraq to alleviate gastrointestinal disorders and oral inflammation; however, its phytochemical composition and pharmacological properties remain scientifically unverified. In this study, we report the first phytochemical profiling and plant-assisted synthesis of copper nanoparticles (CuNPs) using the methanolic extract of *T. trachycarpum* as a natural reducing and stabilizing agent. The synthesized nanoparticles were characterized using UV–Vis spectroscopy, FTIR spectroscopy, X-ray Diffraction (XRD), Transmission Electron Microscopy (TEM), and Energy-Dispersive X-ray Spectroscopy (EDS) analyses, confirming their nanoscale formation, crystallinity, and elemental composition. Gas chromatography–mass spectrometry (GC–MS) identified 22 bioactive metabolites, with methoxsalen (30.91%), triphenylphosphine oxide (12.54%), desulphosinigrin (10.79%), isopimpinellin (6.72%), and α-glyceryl linolenate (6.39%) as the predominant constituents. Both the crude extract and the biosynthesized CuNPs were evaluated for their antimicrobial, antioxidant, and enzyme inhibitory activities. The CuNPs displayed enhanced antimicrobial potency, with MIC values of 250 µg/mL against *Klebsiella pneumoniae* and *Candida albicans*, and 500 µg/mL against *Pseudomonas aeruginosa* and *Staphylococcus epidermidis*. They also exhibited superior antioxidant activity in the 2,2-diphenyl-1-picrylhydrazyl (DPPH), ferric reducing antioxidant power (FRAP), cupric ion reducing antioxidant capacity (CUPRAC), and metal chelating activity (MCA) assays, along with moderate inhibition of key metabolic and neurological enzymes, including acetylcholinesterase and tyrosinase. These findings highlight *T. trachycarpum* as a promising phytochemical source for sustainable nanoparticle synthesis and reveal the multifunctional potential of biosynthesized CuNPs as antioxidant and antimicrobial agents with prospective applications in drug discovery and nanomedicine.

## 1. Introduction

**Figure 1:**
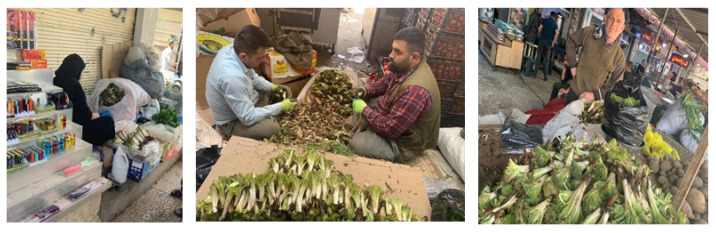
Kurdish vendors offering traditional herbal remedies in local markets (photographs captured by V.S.A.).

Herbal remedies play a fundamental role in traditional medicine within the Kurdistan Region of Iraq, where approximately 133 distinct therapeutic uses have been documented [1]. Many of these applications are medicinal, although some also have cosmetic or ritual significance. Among these uses, plants employed to treat digestive system disorders are widespread, reflecting the high prevalence of such ailments within Kurdish communities (Figure 1) [2,3].

Despite the region’s exceptional botanical diversity, only a limited number of ethnobotanical and phytochemical investigations have been conducted on native flora. Consequently, many traditional uses of these plants remain scientifically unsubstantiated, lacking systematic chemical or pharmacological evaluations [4,5]. The Kurdistan Region, particularly its mountainous areas, hosts a wealth of wild herbs that have long been utilized as natural remedies for numerous health conditions [6]. Traditional healers continue to play a crucial role in local healthcare systems, as herbal medicine often represents the first line of treatment for many diseases. This reliance is especially pronounced among economically disadvantaged populations with limited access to conventional pharmaceuticals [7].

Within this ethnopharmacological framework, *Tordylium trachycarpum* Boiss. (Apiaceae) is a notable medicinal species traditionally used to alleviate stomachaches and stomatitis. Despite its therapeutic relevance, no comprehensive scientific studies have been conducted to elucidate its phytochemical constituents or biological activities. Integrating ethnomedicinal knowledge with contemporary nanotechnology provides an innovative and sustainable approach to discovering new bioactive materials with enhanced pharmacological potential [8].

Over the past decade, green synthesis of metallic nanoparticles using plant extracts has emerged as a promising alternative to conventional physicochemical methods [9]. Unlike chemical or physical routes, which often involve hazardous reagents and high-energy inputs, the plant-mediated approach is more sustainable, cost-effective, and biocompatible [10]. It employs plant-derived biomolecules such as flavonoids, terpenoids, phenolic acids, alkaloids, and proteins as natural reducing, capping, and stabilizing agents [11]. Although solvents such as methanol or hexane are sometimes used in the initial extraction stage, the actual nanoparticle synthesis process remains free of toxic chemicals, aligning with the principles of green chemistry [12]. Among various metallic nanostructures, copper nanoparticles (CuNPs) have attracted considerable attention owing to their broad spectrum of biological and catalytic activities, including antimicrobial, antioxidant, and enzyme inhibitory effects [13].

Nanoparticles (NPs) can be synthesized via chemical, physical, or green (biological) methods [9]. Chemical approaches, including chemical reduction, electrochemical techniques, photochemical reduction, and radiochemical methods using agents such as sodium borohydride or hydrazine, can produce well-defined nanoparticles but are often costly, inefficient, and generate environmentally harmful byproducts [6,9,10]. Physical methods, such as condensation, evaporation, and laser ablation, avoid the use of chemical reagents but require high energy input and expensive equipment [14]. In contrast, green synthesis, particularly plant-mediated methods, offers a sustainable alternative, using extracts that act as both reducing and capping agents to control nanoparticle size, shape, and surface properties [11,15]. Although green-synthesized nanoparticles are often larger than chemically produced ones, they generally exhibit higher biocompatibility, enhanced biofunctionality, and higher yields with minimal environmental impact [8,16].

Green nanotechnology is increasingly favored for nanoparticle fabrication due to its simplicity, low cost, and environmental safety [15]. The nanoscale dimensions of CuNPs confer a high surface-to-volume ratio, which enhances their catalytic and biological reactivity. These features enable CuNPs to interact effectively with microbial cell membranes, resulting in significant antimicrobial potency [17,18]. Beyond biomedical applications, copper nanoparticles are widely utilized in electrocatalysis, photocatalysis, and organic synthesis owing to their favorable physicochemical characteristics, structural stability, and affordability [19].

Plant extracts provide a biologically rich medium that facilitates nanoparticle formation through the synergistic action of various biomolecules. However, achieving precise control over nanoparticle size, shape, and dispersion remains a significant challenge in green synthesis [20]. Previous reports indicate that copper-based nanoparticles exhibit potent antimicrobial properties against various bacterial and fungal strains, including oral pathogens, and can generate reactive oxygen species (ROS), contributing to cytotoxic effects [21,22].

Members of the Apiaceae family are aromatic herbs rich in secondary metabolites, such as coumarins, flavonoids, and terpenoids, which exhibit diverse pharmacological properties, including antimicrobial, antioxidant, and anti-inflammatory effects [23]. *T. trachycarpum* Boiss., a representative of this family, shares the phytochemical characteristics common to many medicinally valuable Apiaceae species.

Given the lack of scientific data on its chemical composition and bioactivity, the present study aimed to, for the first time, investigate the phytochemical profile of the methanolic extract of *T. trachycarpum* using gas chromatography–mass spectrometry (GC–MS). Additionally, this extract was utilized for the green synthesis of copper nanoparticles (CuNPs), employing copper sulfate as a precursor. The resulting nanoparticles and crude extract were subsequently evaluated for their antimicrobial, antioxidant, and enzyme inhibitory activities. This study provides novel insights into the medicinal potential of *T. trachycarpum* and underscores its significance as a sustainable source for nanomaterial synthesis within the growing field of green nanotechnology.

## 2. Materials and Methods

### 2.1. Materials and Instrumentations

All chemicals and solvents (HPLC grade) were obtained from commercial suppliers and used without further purification. Analytical thin-layer chromatography (TLC) was performed on silica gel 60 F254 plates, visualized under UV light (254 nm), or immersed in an appropriate staining reagent. Solid-phase extraction was performed using a C18 cartridge (Discovery DSC-18 SPE Tube, 10 g bed weight, 60 mL volume; Supelco, Bellefonte, PA, USA). Mueller–Hinton agar and Sabouraud dextrose agar were purchased from Thermo Fisher Scientific (Waltham, MA, USA). Fourier-transform infrared spectroscopy (FT-IR) spectra were recorded using an IR-Affinity-1 SHIMADZU spectrometer (Kyoto, Japan), and UV–Vis spectra were obtained with a UV–Vis Spectrophotometer AE/S60 (AE-S60, A&E Lab Instruments Co., Ltd., Guangzhou, China), equipped with a 1.0 cm quartz cuvette was used at Salahaddin University–Erbil, Kurdistan Region, Iraq. X-ray diffraction (XRD) analysis was performed using a Bruker D2 Phaser diffractometer (380 eV), (D2 Phaser, Bruker AXS GmbH, Karlsruhe, Germany), and Energy-dispersive X-ray spectroscopy (EDS) was performed using a QUANTAX EDS silicon drift detector (Bruker AXS GmbH, Karlsruhe, Germany) at Tehran University, Iran. GC–MS analysis was carried out on a Thermo Scientific DSQII single quadrupole system (Trace GC Ultra gas chromatograph, TriPlus autosampler; Thermo Fisher Scientific, Waltham, MA, USA) at Centro Grandi Strumenti, University of Pavia, Italy.

### 2.2. Plant Collection

**Figure 2:**
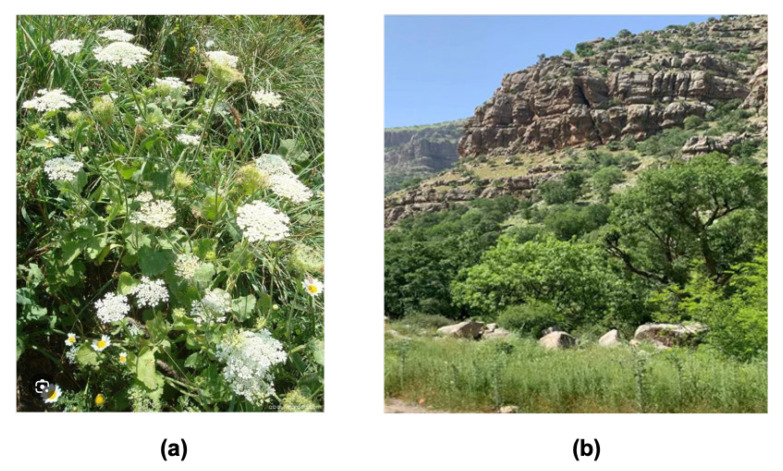
(**a**) *Tordylium trachycarpum* plant; (**b**) Barzan Town, Kurdistan Region of Iraq (photographs captured by V.S.A.).

Aerial parts of *T. trachycarpum* were collected near Barzan town, Erbil, Kurdistan Region of Iraq, during the flowering stage in April 2024. The species was botanically identified by Dr. Serwan T. Al-Dabbagh at Salahaddin University–Erbil, and a voucher specimen (No. 7631) was deposited in the Herbarium of the Department of Biology, College of Science, Salahaddin University–Erbil. Freshly collected aerial parts were air-dried at room temperature in the shade under active ventilation (Figure 2).

### 2.3. Extraction Process and Preliminary Chromatographic Purification of the Methanolic Extract

The air-dried aerial parts of *T. trachycarpum* were ground into a fine powder using a laboratory mill. For extraction, 50 g of powdered plant material was initially defatted with 250 mL of n-hexane to remove nonpolar constituents. The residue was subsequently extracted with 250 mL of methanol and subjected to ultrasonication for 30 min to enhance solvent penetration and release bioactive compounds. Following sonication, the mixture was macerated overnight at room temperature and filtered. The filtrate was then concentrated under reduced pressure using a rotary evaporator to obtain the crude methanolic extract.

**Figure 3:**
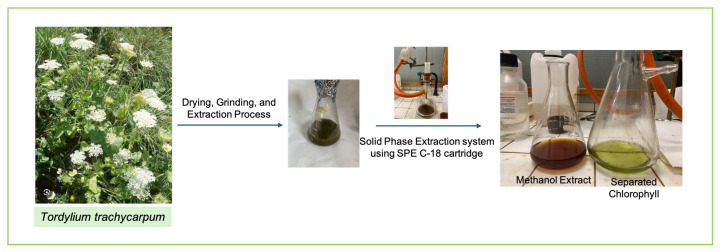
Extraction Process and Preliminary Chromatographic Purification of the Methanolic Extract.

To eliminate chlorophylls and residual lipophilic impurities, the methanolic extract (1 g) was purified by solid-phase extraction (SPE) using a C18 cartridge (Discovery DSC-18 SPE Tube, 10 g bed weight, 60 mL volume, Supelco, Bellefonte, PA, USA). The cartridge was preconditioned sequentially with 50 mL of methanol, followed by 50 mL of distilled water. The sample was dissolved in a methanol–water mixture (90:10, *v*/*v*) under ultrasonic agitation for 30 min and then loaded onto the cartridge. Elution was performed with 250 mL of methanol–water (90:10, *v*/*v*), and the eluate was concentrated to dryness under reduced pressure (Figure 3) [3]. The purified extract was stored in airtight containers at 4 °C until further analysis.

### 2.4. Synthesis of Copper Nanoparticles

**Figure 4:**
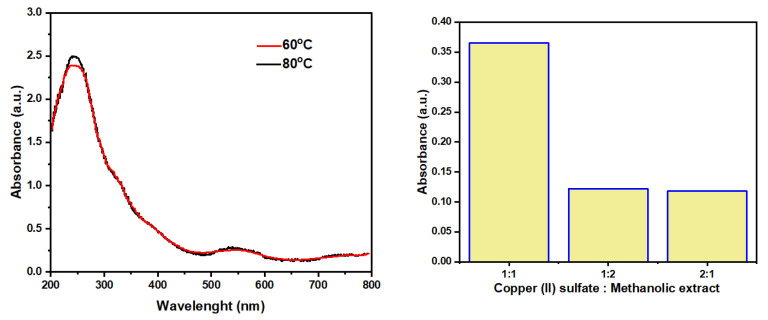
Optimization parameters in the copper nanoparticle synthesis process.

**Figure 5:**
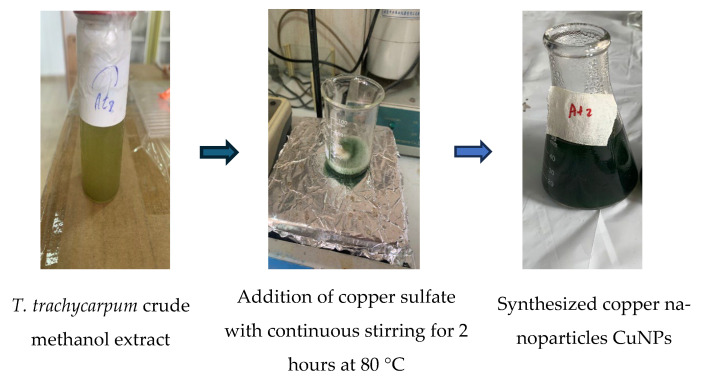
Schematic representation of the copper nanoparticle synthesis process.

Copper nanoparticles (CuNPs, Tt2) were synthesized using a plant-assisted green synthetic approach. Based on preliminary optimization experiments and supporting literature on plant-mediated nanoparticle synthesis, the reaction parameters concentration, temperature, reaction time, and volume ratio were systematically evaluated to achieve efficient and stable nanoparticle formation. Equal volumes of 1% (*w*/*v*) copper (II) sulfate solution and 1% (*w*/*v*) *T. trachycarpum* methanolic extract were mixed in a 1:1 ratio under continuous stirring. Preliminary trials with varying volume ratios (1:1, 2:1, and 1:2) and temperatures (60 °C and 80 °C) indicated that the 1:1 ratio at 80 °C for 2 h produced the most uniform and stable nanoparticles (Figure 4). This condition was selected as optimal because 80 °C provides a suitable balance between the reduction kinetics of Cu^2+^ ions and the thermal stability of phytochemicals, minimizing their degradation while enhancing the reaction rate. The synthesis was performed in dark, closed containers to prevent photo-oxidation and maintain the metallic state of the CuNPs. A visible color change from olive-brown to dark green indicated successful nanoparticle formation (Figure 5). The resulting colloidal suspension was centrifuged at 10,000 rpm for 15 min, washed three times with distilled water to remove unreacted residues, and stored in airtight containers at 4 °C until further characterization and biological evaluation [24].

### 2.5. Antimicrobial Assay

Antimicrobial activity was evaluated using the agar well diffusion method against *Escherichia coli* (NCIMB12454), *Pseudomonas aeruginosa* (NCIMB50126), *Staphylococcus epidermidis* (NCIMB50110), *Klebsiella pneumoniae* (NCIMB13218), *Staphylococcus aureus* (NCIMB9518), and *Candida albicans* (NCIMB8054). Mueller–Hinton and Sabouraud dextrose agars were used for bacteria and yeast, respectively. The microorganisms were prepared by growing overnight, and the turbidity was maintained at 0.5 MacFarland (nearly 10~8 colony-forming units per milliliter for bacteria and 10~5 colony-forming units per milliliter for fungi). An individual was inoculated with a microbial cell suspension using a cotton swab. Wells (6 mm) were formed by using sterile tips, and each of them was filled with 100 µL of plant extract solution (500 µg/mL) or synthesized copper nanoparticles (500 µg/mL). Furthermore, ampicillin and fluconazole were used as positive controls for bacteria and fungi, respectively, and ethanol was used as a negative control. The plates were incubated for 24 h at 37 °C for bacteria, followed by 72 h incubation at room temperature for yeast. After the specific times for layer growth of the microorganisms, the zone of inhibition was measured using a ruler.

### 2.6. Minimum Inhibitory Concentration (MIC)

MIC values were determined using the twofold serial dilution method in 96-well microplates. Stock solutions of CuNPs (Tt2) and plant extract (Tt1) were prepared at 2000 µg/mL and serially diluted (2000, 1000, 500, 250, 125 µg/mL). Forty microliters of microbial suspension were added to wells containing 100 µL of each sample. Positive and negative controls were included, and the plates were incubated at 37 °C for 24 h. Growth inhibition was assessed by comparing the wells with the controls.

### 2.7. Antioxidant Assay

The antioxidant potential of the crude extract (Tt1) and biosynthesized copper nanoparticles (CuNPs, Tt2) was evaluated using multiple in vitro assays, including DPPH and ABTS radical scavenging, cupric ion reducing antioxidant capacity (CUPRAC), ferric reducing antioxidant power (FRAP), metal-chelating activity (MCA), and phosphomolybdenum assay (PMA), according to previously reported methods [25,26].

DPPH radical scavenging assay: 1 mL of the sample (1 mg/mL) was added to 1 mL of 0.1 mM DPPH solution, and the mixture was incubated in the dark for 30 min. Absorbance was measured at 517 nm.

ABTS assay: 1 mL of the sample (1 mg/mL) was mixed with 1 mL of ABTS•^+^ solution and incubated for 30 min. Absorbance was recorded at 734 nm.

CUPRAC assay: 0.5 mL of the sample (1 mg/mL) was reacted with CUPRAC reagent and incubated for 30 min. Absorbance was measured at 450 nm.

FRAP assay: 0.1 mL of the sample (1 mg/mL) was mixed with FRAP reagent and incubated at room temperature for 30 min. Absorbance was measured at 593 nm.

Metal-chelating activity (MCA): 2 mL of the sample (1 mg/mL) was mixed with Fe^2+^ solution, and chelation was assessed at 562 nm.

Phosphomolybdenum assay (PMA): 0.3 mL of the sample (1 mg/mL) was incubated with PMA reagent, and absorbance was measured at 695 nm.

All assays were performed in triplicate, and the results were expressed as the mean ± standard deviation.

### 2.8. Total Phenolic and Flavonoid Contents Assay

For phenolic content, 1 mL of diluted Folin–Ciocalteu reagent (1:9 *v*/*v*) was combined with 0.25 mL of sample extract. After 3 min of shaking, 0.75 mL of 1% Na_2_CO_3_ solution was added. Following 2 h of incubation at room temperature, absorbance was measured at 760 nm. The results were expressed as mg gallic acid equivalents (mg GAE/g extract) [25].

For total flavonoid content, 1 mL of the extract was mixed with 2% aluminum chloride in methanol. A blank was prepared using methanol. After 10 min at room temperature, absorbance was measured at 415 nm. Total flavonoids were expressed as mg rutin equivalent (mg RE/g extract) [25].

### 2.9. Enzyme-Inhibitory Assay

The inhibitory activities of Tt1 and Tt2 against acetylcholinesterase (AChE), butyrylcholinesterase (BChE), tyrosinase, α-amylase, and α-glucosidase were evaluated according to established protocols [26]. Sample solutions (1 mg/mL) were prepared in the respective assay buffers. Reactions were initiated by adding enzyme solutions to the wells containing the test samples. Incubations were carried out under the conditions described in the reference methods. Enzyme activity was measured spectrophotometrically at the following wavelengths: AChE and BChE, 405 nm; tyrosinase, 492 nm; α-amylase, 540 nm; α-glucosidase, 405 nm. Percentage inhibition was calculated relative to the negative controls. Ethanol or buffer-only solutions were used as negative controls, whereas standard inhibitors, including galantamine for cholinesterases and kojic acid for tyrosinase, served as positive controls. All experiments were performed in triplicate, and the data are presented as the mean ± standard deviation.

### 2.10. Gas Chromatography–Mass Spectrometry (GC–MS) Analysis

GC-MS analyses were carried out using a Thermo Scientific Focus GC system coupled with a DSQ mass spectrometer detector (Trace GC Ultra gas chromatograph, TriPlus autosampler; Thermo Fisher Scientific, Waltham, MA, USA), operating in electron-impact (EI) ionization mode at 70 eV. Separation was achieved using a nonpolar Agilent J&W HP-5 fused silica capillary column (30 m × 0.25 mm inner diameter, 0.25 µm film thickness, 5% phenylmethylpolysiloxane; Agilent Technologies Italia S.p.A., Cernusco sul Naviglio, MI, Italy). Helium (He) was used as the carrier gas at a constant flow rate of 1.0 mL/min.

The methanolic extract (Tt1) sample was prepared by dissolving the extract in methanol to a final concentration of 1 mg/10 mL, and 1 µL of the solution was injected into the system in split mode (split ratio 20:1). The GC oven temperature was initially set at 60 °C for 1 min, followed by a temperature ramp of 5 °C/min up to 260 °C, where it was held for 5 min. The injector and transfer-line temperatures were both maintained at 250 °C, and the acquisition mass range was set to *m*/*z* 41–350 amu. Data acquisition and analysis were performed using MSD ChemStation software (E.02.02). The identification of compounds was carried out by comparing the obtained mass spectra and retention indices with the NIST Mass Spectral Library (NIST 98) and literature reports [27]. The relative percentages of each constituent were calculated based on peak area normalization.

### 2.11. Phytochemical Profiling of the Post-Synthesis Supernatant

To assess the consumption of bioactive compounds during nanoparticle formation, the supernatant obtained after CuNP separation was analyzed for total phenolic and flavonoid content using the same procedures described in Section 2.8.

### 2.12. Statistical Analysis

All experiments were performed in triplicate (*n* = 3). Data are presented as the mean ± standard deviation (SD). Statistical significance was determined using one-way ANOVA, followed by Tukey’s post hoc test and Student’s *t*-test. Image data were processed and analyzed using ImageJ v1.54f (NIH, Bethesda, MD, USA), a Java-based open-source image analysis software supporting multi-format image import, stacks, ROI-based measurements, thresholding, object quantification and statistical output. Data analysis and graphing were performed using OriginPro 2024 (OriginLab Corporation, Northampton, MA, USA), a Windows-based scientific data analysis and plotting software that provides tools for curve fitting, regression, descriptive statistics, and high-quality 2D/3D graphing.

## 3. Results and Discussion

### 3.1. Phytochemical Analysis

Traditionally, local inhabitants have used *T. trachycarpum* to relieve stomachaches and stomatitis by drying the plant at room temperature and then dissolving it in water before use. These therapeutic properties are likely attributed to the presence of various phytochemicals and secondary metabolites.

In this study, the aerial parts of *T. trachycarpum* were initially defatted using n-hexane to remove nonpolar constituents. The extraction was facilitated by ultrasonic treatment, which enhanced solvent penetration and reduced extraction time by generating ultrasound waves. The resulting residue was subsequently extracted with methanol under identical conditions to isolate the active polar components.

**Figure 6:**
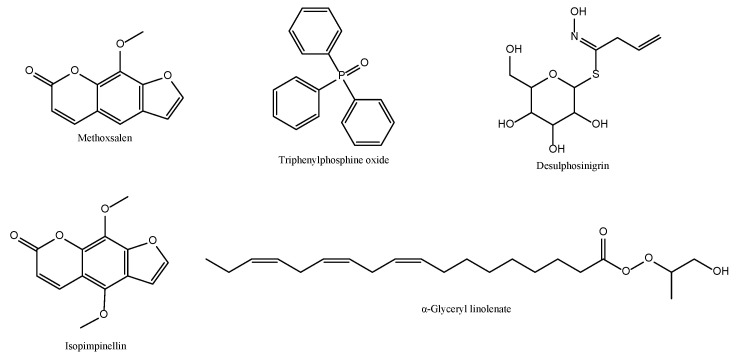
Primary chemical constituents are present in the methanolic extract of *T. trachycarpum*.

Gas chromatography–mass spectrometry (GC–MS) analysis of the methanolic extract identified 22 phytochemical constituents (Table 1). The major identified compounds included methoxsalen (30.91%), triphenylphosphine oxide (12.54%), and desulphosinigrin (10.79%), followed by isopimpinellin (6.72%) and α-glyceryl linolenate (6.39%) (Figure 6).

These bioactive compounds, particularly furanocoumarins, such as methoxsalen and bergapten, are well known for their antimicrobial, antioxidant [28], and anti-inflammatory [29] activities, supporting the plant’s traditional medicinal applications. The diversity and abundance of such metabolites highlight the pharmacological potential of *T. trachycarpum* and justify further investigation into its biological properties.

The chemical structure of the main chemical constituents available in the plant mentioned above and detected by GC-MS is presented in Figure 4.

### 3.2. Characterization of Synthesized Copper Nanoparticles

**Figure 7:**
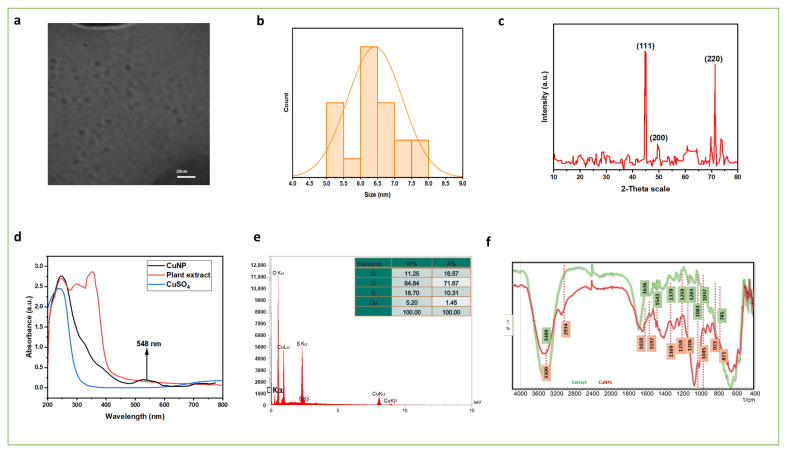
Complete characterization of the synthesized CuNPs including (**a**) TEM image of green synthesized Cu nanoparticles, (**b**) Size distribution of Cu nanoparticles, (**c**) XRD pattern for green synthesis Cu, (**d**) Uv-Vis spectrophotometry to study optical properties of Cu nanoparticles, (**e**) EDS analysis to study formed Cu nanoparticles, (**f**) FTIR spectrum to study the functional groups on surface of Cu.The red dotted lines mark the FTIR peak positions of the CuNPs.

Transmission electron microscopy (TEM) analysis revealed that the biosynthesized copper nanoparticles (CuNPs) were predominantly spherical and well dispersed, with an average particle size of 6.25 nm and a narrow distribution of 5–8 nm (Figure 7a,b) [30]. The uniform morphology and limited aggregation observed confirmed the effectiveness of phytochemical capping agents in controlling particle growth and ensuring colloidal stability.

X-ray diffraction (XRD) analysis further confirmed the crystalline nature of the CuNPs, showing distinct diffraction peaks at 2θ = 44.8°, 50.1°, and 72.8°, corresponding to the (111), (200), and (220) planes of face-centered cubic (FCC) copper, consistent with standard JCPDS data (Figure 7c) [31]. No additional peaks corresponding to the CuO or Cu_2_O phases were detected, suggesting the successful synthesis of phase-pure metallic copper. A slight broadening of the diffraction peaks was observed, attributable to the small crystallite size and minor nanoparticle aggregation, a phenomenon commonly reported in plant-mediated nanoparticle syntheses and often associated with variations in particle size distribution or partial agglomeration [31,32,33]. The CuNPs were stored in airtight containers protected from light and heat at both room temperature and 4 °C for 7 days. No visible aggregation or precipitation was observed during this period, indicating good colloidal stability due to effective phytochemical capping. Additionally, upon storage, the CuNPs retained their biological activity when reused for antibacterial and antifungal assays, confirming that their structural integrity and functionality were retained. The observed uniformity in particle size and effective phytochemical capping suggests that the CuNPs are likely to exhibit good colloidal stability over time, minimizing aggregation under appropriate storage conditions and maintaining their functional properties for practical applications [34].

Energy-dispersive X-ray spectroscopy (EDS) confirmed copper as the dominant element, with oxygen detected due to minor surface oxidation. Trace carbon and sulfur signals likely originated from phytochemical residues acting as natural stabilizers. The corresponding elemental composition (wt%) was C: 11.25, O: 64.84, S: 18.70, and Cu: 5.20 (Figure 7d) [35].

UV–visible spectroscopy revealed a distinct surface plasmon resonance (SPR) absorption band at 548 nm, confirming the successful formation of colloidal CuNPs with good dispersion (Figure 7e). Notably, the SPR band exhibited slight broadening, which is commonly attributed to nanoscale crystallite dimensions and possible surface effects or partial nanoparticle aggregation. This phenomenon has been widely documented for metal nanoparticles synthesized via plant-mediated approaches, where heterogeneous particle size distribution and interactions between phytoconstituents and nanoparticle surfaces can induce spectral broadening. The literature reports further support that SPR broadening arises from the combined effects of quantum confinement, capping agent influence, and interparticle aggregation during nanoparticle formation. Accordingly, the broad UV–vis absorption band observed in this study is consistent with previous findings and complements the TEM-based particle size distribution results [35,36,37].

Fourier-transform infrared (FTIR) spectroscopy revealed apparent spectral differences between the crude extract and the synthesized CuNPs (Figure 7f). A broad O–H/N–H stretching band at 3309 cm^−1^ indicated hydrogen-bonding interactions, while a shift in the amide and carbonyl stretching region from 1636 to 1659 cm^−1^ confirmed coordination between phytochemical ligands and copper ions. The emergence of new peaks at 923 and 871 cm^−1^ further supports the reduction and stabilization of copper nanoparticles by functional biomolecules [38]. The FTIR spectra revealed characteristic absorption bands corresponding to hydroxyl (O–H), amine (N–H), and carbonyl (C=O) groups, suggesting their active participation in Cu–ligand coordination and surface capping [39]. These groups likely chelate copper ions through electron donation, stabilizing the nanoparticles and preventing aggregation [38].

Together, these results confirmed the successful green synthesis of stable, crystalline, and uniformly dispersed CuNPs, mediated by the phytochemicals of *T. trachycarpum*, with polyphenols and flavonoids playing crucial roles as natural reducing and capping agents. The structural uniformity, effective capping, and minimal aggregation indicate that these CuNPs are expected to maintain their stability and functional properties over time under suitable storage conditions.

### 3.3. Antibacterial and Antifungal Activity

**Figure 8:**
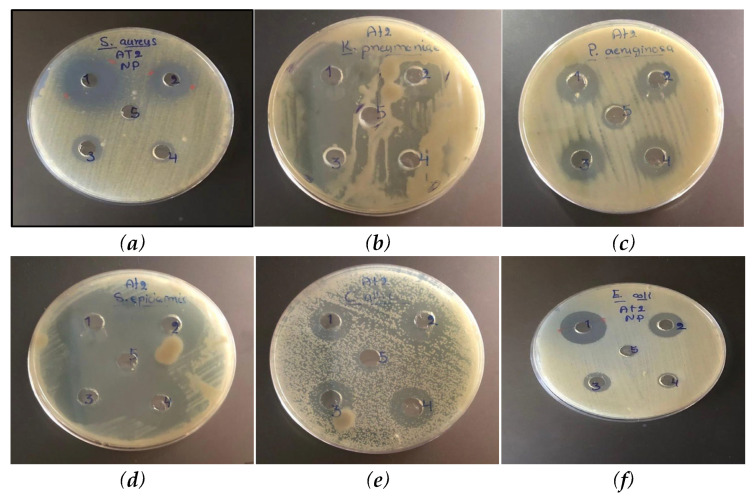
Antimicrobial activity of the synthesized copper nanoparticles (Tt2) against *Staphylococcus aureus* (**a**), *Klebsiella pneumoniae* (**b**), *Pseudomonas aeruginosa* (**c**), *Staphylococcus epidermidis* (**d**), *Candida albicans* (**e**), and *Escherichia coli* (**f**).

The antimicrobial potential of the methanolic extract of *T. trachycarpum* (Tt1) and its biosynthesized copper nanoparticles (Tt2) was systematically evaluated against a panel of bacterial and fungal pathogens using the agar well diffusion method. The antimicrobial efficacy was expressed as the diameter of the inhibition zones (mm) and is summarized in Table 2, while representative inhibition patterns are illustrated in Figure 8.

The results revealed that the copper nanoparticle formulation (Tt2) exhibited significantly greater antimicrobial activity than the crude methanolic extract (Tt1), which showed only weak to moderate inhibition. The most pronounced inhibition zones were recorded against *Staphylococcus epidermidis* and *Klebsiella pneumoniae* (5 mm) as well as *Candida albicans* (3.4 mm), indicating broad-spectrum antibacterial and antifungal potential.

The enhanced antimicrobial efficacy of Tt2 may be attributed to the generation of reactive oxygen species (ROS), leading to oxidative stress, membrane damage, and subsequent cell death. Additionally, the release of Cu^+^ and Cu^2+^ ions can disrupt microbial membranes, interfere with enzymatic functions, and alter cellular metabolism [40,41]. These findings are consistent with previous studies demonstrating the superior antibacterial performance of plant-mediated copper nanoparticles [42,43]. The observed inhibition of resistant bacterial strains further highlights the potential of *T. trachycarpum*-derived Cu NPs as effective natural antimicrobial agents.

### 3.4. Minimum Inhibitory Concentration (MIC)

The minimum inhibitory concentration (MIC) assay further demonstrated the superior antimicrobial efficacy of the biosynthesized copper nanoparticles (Tt2) compared to the crude methanolic extract (Tt1). The CuNPs exhibited markedly lower MIC values across most tested microbial strains, indicating greater potency and a broader antimicrobial spectrum. Specifically, the MIC values of Tt2 against *Klebsiella pneumoniae* and *Candida albicans* were 250 µg/mL. In contrast, those against *Pseudomonas aeruginosa* and *Staphylococcus epidermidis* were 500 µg/mL, all of which were substantially lower than the corresponding MIC values for the crude extract (Table 3). These findings confirmed that incorporating copper nanoparticles significantly enhanced the antimicrobial activity of bioactive constituents of *T. trachycarpum*.

The enhanced biological performance of Tt2 can be attributed to the synergistic action of copper ions and the phytochemical constituents of *T. trachycarpum*, which together improve the crude extract’s antimicrobial activity [44]. The MIC results further demonstrated a concentration-dependent inhibitory pattern, with higher concentrations producing more substantial antimicrobial effects. This may be related to the presence of phenolic and flavonoid compounds, which are known to disrupt microbial membranes and inhibit enzyme activity [45,46].

Moreover, the nanoscale size and high surface-to-volume ratio of the synthesized CuNPs likely facilitate greater interaction with microbial cell walls, enhancing penetration and reactivity [18]. Consequently, the lower MIC values observed for Tt2 reflect their superior bioactivity and confirm that both phytochemical richness and nanoparticle formation play key roles in boosting antimicrobial efficiency and reducing microbial resistance.

### 3.5. Total Flavonoid and Phenolic Composition

The total phenolic and flavonoid content of the methanolic extract (Tt1) and its biosynthesized copper nanoparticles (Tt2) derived from *T. trachycarpum* are presented in Table 4. The methanolic extract exhibited a high total phenolic content of 26.22 ± 0.50 mg GAE/g extract and a total flavonoid content of 46.90 ± 0.56 mg QE/g extract, indicating the richness of *T. trachycarpum* in antioxidant phytochemicals.

To further assess the role of these compounds in nanoparticle formation, the total phenolic and flavonoid contents were also measured in the supernatant obtained after CuNP synthesis [47]. A significant reduction in both parameters was observed compared to the crude extract, suggesting that polyphenolic and flavonoid molecules were actively involved in the decrease of Cu^2+^ ions and the stabilization of the resulting nanoparticles [10]. These findings confirm that the phenolic and flavonoid constituents act as natural reducing and capping agents, facilitating nanoparticle biosynthesis through electron donation and complexation with metal ions.

This observation aligns with previous studies reporting similar mechanisms, where plant-derived phenolics and flavonoids participate directly in the bioreduction and stabilization of metallic nanoparticles [48]. The high concentration of these phytochemicals in *T. trachycarpum* may therefore contribute not only to efficient nanoparticle synthesis but also to the enhanced antioxidant and antimicrobial activities observed for Tt2.

The elevated levels of these secondary metabolites highlight the phytochemical richness of *T. trachycarpum*, supporting its traditional medicinal use. Given their well-established redox potential and ability to neutralize reactive oxygen species [27], these compounds likely play a central role in the pronounced antioxidant properties demonstrated in subsequent assays.

### 3.6. Antioxidant Capacity

To comprehensively evaluate the antioxidant potential of *T. trachycarpum* methanolic extract (Tt1) and its copper nanoparticles (Tt2), several complementary antioxidant assays were conducted, including DPPH, ABTS, CUPRAC, FRAP, MCA, and PMA tests. These assays collectively assess radical-scavenging ability, reducing power, and metal-chelating capacity.

As shown in Table 5, the Tt2 nanoparticles demonstrated superior antioxidant activity in most assays compared to the crude methanolic extract (Tt1). In particular, Tt2 exhibited higher reducing power in the FRAP (113.15 ± 1.22 mg TE/g) and CUPRAC (82.11 ± 0.72 mg TE/g) assays than Tt1 (103.52 ± 8.16 mg TE/g and 63.04 ± 1.87 mg TE/g, respectively). Similarly, in the DPPH assay, Tt2 showed a significantly greater radical scavenging capacity (88.42 ± 0.96 mg TE/g) than Tt1 (61.42 ± 1.30 mg TE/g). Metal chelating activity (MCA) also followed this trend, with Tt2 recording 11.42 ± 1.44 mg EDTAE/g compared to 6.94 ± 0.69 mg EDTAE/g for Tt1, indicating a more substantial metal-binding potential.

Conversely, in the ABTS and phosphomolybdenum (PMA) assays, Tt1 showed a slightly higher antioxidant performance than Tt2. The ABTS radical scavenging values were 135.18 ± 2.84 mg TE/g for Tt1 and 128.89 ± 4.96 mg TE/g for Tt2, while the total antioxidant capacity determined by PMA was higher in Tt1 (2.56 ± 0.12 mmol TE/g) compared to Tt2 (1.38 ± 0.03 mmol TE/g).

Overall, these results indicate that green-synthesized Cu nanoparticles (Tt2) possess enhanced antioxidant capabilities in most assays, particularly those involving electron transfer and metal ion chelation. The improved activity of Tt2 can be attributed to the synergistic interaction between copper ions and the phytochemical constituents of *T. trachycarpum*, which facilitates more efficient redox reactions and free radical neutralization. The slightly higher activity of Tt1 in the ABTS and PMA tests may reflect the presence of soluble antioxidant compounds that remain more active in the crude extract.

These findings highlight the potential of *T. trachycarpum*-derived Cu nanoparticles as effective antioxidant agents, supporting their suitability for biomedical and pharmaceutical applications.

### 3.7. Enzyme-Inhibitory Assay

The enzyme inhibition potential of *T. trachycarpum* methanolic extract (Tt1) and its copper nanoparticles (Tt2) was evaluated against five key enzymes, acetylcholinesterase (AChE), butyrylcholinesterase (BChE), tyrosinase, α-amylase, and α-glucosidase, to explore their possible neuroprotective and antidiabetic properties (Table 6).

The methanolic extract (Tt1) demonstrated moderate inhibitory activity against AChE (2.29 ± 0.20 mg GALAE/g) and weak activity against BChE (0.89 ± 0.03 mg GALAE/g). At the same time, the nanoparticle formulation (Tt2) exhibited lower inhibition toward AChE (1.78 ± 0.09 mg GALAE/g) and was inactive against BChE. The decline in cholinesterase inhibition following nanoparticle formation may be attributed to interactions between copper ions and polar phytochemicals, possibly altering the binding affinity for enzyme-active sites [49].

In contrast, both samples showed substantial tyrosinase inhibitory activity, with Tt2 (52.62 ± 1.96 mg KAE/g) outperforming Tt1 (46.96 ± 3.18 mg KAE/g). This enhancement indicates that nanoparticle formation improves the interaction between phenolic or flavonoid components and the copper-containing catalytic center of the enzyme, resulting in more efficient enzyme–inhibitor complex formation [50]. Tyrosinase inhibition is of particular interest due to its relevance in managing hyperpigmentation disorders and neurodegenerative conditions associated with oxidative stress [51].

Regarding carbohydrate-hydrolyzing enzymes, both extracts exhibited relatively weak α-amylase and α-glucosidase inhibition. Tt1 showed moderate α-amylase inhibition (0.57 ± 0.01 mmol ACAE/g) and mild α-glucosidase inhibition (1.12 ± 0.02 mmol ACAE/g), whereas Tt2 showed reduced α-amylase activity (0.35 ± 0.02 mmol ACAE/g) and was inactive against α-glucosidase. The lower inhibitory effect of Tt2 in these assays suggests that the phenolic compounds responsible for glycosidase inhibition may have undergone structural modifications or become less accessible after nanoparticle synthesis [52].

The enzyme-inhibitory activity of *T. trachycarpum*–derived copper nanoparticles (CuNPs), particularly against tyrosinase and acetylcholinesterase (AChE), can be attributed to the strong affinity of copper ions and surface-bound phytochemicals for the catalytic residues of these enzymes. The CuNPs are likely to interact with amino acid residues located at or near the active sites, forming coordination bonds that modify the local electronic environment and alter enzyme conformation. Such interactions may lead to competitive, non-competitive, or mixed-type inhibition, depending on whether the nanoparticles obstruct substrate binding or induce conformational changes within the catalytic pocket [53]. In addition, phenolic and flavonoid molecules adsorbed on the nanoparticle surface may enhance inhibition synergistically through hydrogen bonding or π–π stacking interactions with aromatic residues [54]. Comparable metal nanoparticle–enzyme interactions have been shown to impair enzyme catalysis via both steric hindrance and oxidative stress mechanisms [55]. Overall, the combined contribution of the metallic core and phytochemical capping agents accounts for the potent and selective inhibitory behavior of the CuNPs, highlighting their potential as multifunctional bioactive nanomaterials.

These results further indicate that while the methanolic extract of *T. trachycarpum* exhibits broad enzyme-inhibitory activity, its copper nanoparticle derivative shows selective enhancement of tyrosinase inhibition. This suggests that nanoparticle formation modulates bioactivity in an enzyme-specific manner—strengthening interactions with certain metalloproteins, such as tyrosinase—while reducing the affinity for other enzymatic targets. Such differential inhibition patterns emphasize the promise of *T. trachycarpum*–based nanomaterials for targeted biomedical and cosmetic applications. The improved biological performance of *T. trachycarpum*–derived CuNPs can be further rationalized through mechanistic insights into their molecular interactions with enzymes and oxidative pathways.

### 3.8. Mechanistic Discussion

The enhanced biological performance of *T. trachycarpum* methanolic extract and its copper nanoparticles (CuNPs) can be rationalized through their underlying molecular mechanisms and structure–activity relationships (SAR).

The biosynthesized CuNPs (Tt_2_) exhibited superior antioxidant and antimicrobial activity compared to the crude extract (Tt_1_), which can be attributed to the synergistic interplay between copper ions and phytoconstituents identified in the GC–MS profile [56]. Major compounds such as methoxsalen, bergapten, isopimpinellin, and α-glyceryl linolenate possess aromatic and oxygenated functional groups capable of reducing Cu^2+^ to Cu^0^ and simultaneously stabilizing the resulting nanoparticles via surface adsorption. These phytochemicals serve as both reducing and capping agents, controlling the particle size, morphology, and surface charge key parameters that govern nanoparticle reactivity and bioactivity [31,35].

The antimicrobial activity of CuNPs arises mainly from their interaction with microbial membranes and the induction of oxidative stress [20]. Upon contact, CuNPs adhere to negatively charged cell walls, releasing Cu^2+^ ions that penetrate the cytoplasm, generate reactive oxygen species (ROS), and disrupt essential biomolecules, including lipids, proteins, and DNA [57]. These processes, combined with the binding of Cu^2+^ ions to enzyme thiol groups and interference with key metabolic enzymes, collectively lead to cellular damage and death, which is consistent with previous reports on metal nanoparticle-induced oxidative stress and membrane disruption [28,29,30,31]. The observed inhibition of *Staphylococcus epidermidis* and *Candida albicans* supports this mechanism.

In antioxidant assays, the CuNPs displayed intense redox cycling, facilitated by Cu^2+^/Cu^+^ interconversion at the nanoparticle surface. The phytochemicals surrounding the CuNPs further contribute through hydrogen atom or electron donation, neutralizing free radicals, and enhancing total reducing power [58]. This synergistic metal–phytochemical interaction explains the higher CUPRAC, FRAP, and DPPH activities of Tt_2_ compared to Tt_1_ [43].

From an SAR perspective, compounds containing conjugated aromatic systems and hydroxyl or methoxy substituents (e.g., methoxsalen, bergapten, and α-glyceryl linolenate) exhibit strong electron-donating properties, facilitating effective metal chelation and electron-transfer reactions [59]. Their incorporation into the CuNP surface enhances both stability and biological activity by improving redox potential and ROS modulation [60].

Overall, the combined effects of phytochemical functionality, metal ion dynamics, and surface chemistry account for the potent antioxidant and antimicrobial behavior of *T. trachycarpum*-derived CuNPs. These findings suggest that green-synthesized CuNPs exert biological effects through coordinated ROS generation, membrane perturbation, and enzyme inhibition, making them promising candidates for the development of novel bioactive materials in antimicrobial and oxidative stress–related drug discovery.

## 4. Conclusions

This study demonstrates a sustainable and plant-assisted approach for the green synthesis of copper nanoparticles (CuNPs) using the methanolic extract of *T. trachycarpum* (Apiaceae) as a natural reducing and stabilizing agent. Phytochemicals such as methoxsalen, triphenylphosphine oxide, desulphosinigrin, isopimpinellin, and α-glyceryl linolenate effectively facilitated the formation of stable crystalline CuNPs, as confirmed by UV–Vis, FTIR, XRD, TEM, and EDS analyses. Compared with conventional chemical synthesis, this plant-mediated route offers a greener, cost-effective, and biocompatible alternative, eliminating the need for hazardous reagents. The biosynthesized CuNPs exhibited vigorous antimicrobial activity against *Staphylococcus aureus* and *Candida albicans* and enhanced antioxidant performance in DPPH, FRAP, CUPRAC, and MCA assays, alongside moderate inhibitory effects against acetylcholinesterase and tyrosinase. These multifunctional properties, likely driven by metal–enzyme coordination and oxidative stress modulation, underscore the potential of *T. trachycarpum*-derived CuNPs as promising candidates for biomedical and pharmaceutical applications within the framework of sustainable nanotechnology.

## Figures and Tables

**Table 1:** Phytochemical constituents present in *T. trachycarpum* detected by GC-MS.

RT	% Area	Phytochemical Constituent	Molecular Formula	Cas No.
2.49	2.09	2,2-Dimethoxybutane	C_6_H_14_O_2_	3453-99-4
3.83	0.17	1,2-Hydrazinedicarboxamide	C_2_H_6_N_4_O_2_	110-21-4
5.8	1.17	Glycerin	C_3_H_8_O_3_	56-81-5
8.61	0.64	1,2,3-Propanetriol, 1-acetate	C_5_H_10_O_4_	106-61-6
9.9	1.27	Pyranone	C_6_H_8_O_4_	28564-83-2
10.83	5.07	Octanoic acid	C_8_H_16_O_2_	124-07-2
12.53	2.6	α-Monoacetin	C_5_H_10_O_4_	106-61-6
16	2.89	4-Methylmannitol	C_7_H_16_O_6_	130073
18.68	4.7	1,4-Di-O-acetyl-2,3,5-tri-O-methylribitol	C_12_H_22_O_7_	84925-40-6
19.46	0.63	2,4-Di-tert-butylphenol	C_14_H_22_O	96-76-4
**21.15**	**10.79**	**Desulphosinigrin**	**C_10_H_17_NO_6_S**	**5115-81-1**
22.57	3.34	D-Melezitose	C_18_H_32_O_16_	597-12-6
25.53	1.41	1-Heptatriacotanol	C_16_H_32_O_2_	105794-58-9
25.84	1.32	Octanoic acid, octyl ester	C_16_H_32_O_2_	2306-88-9
26.81	0.77	Isopsoralen	C_11_H_6_O_3_	523-50-2
29.16	0.72	R-1 Methanandamide	C_23_H_39_NO_2_	157182-49-5
29.55	2.21	Palmitic acid	C_16_H_32_O_2_	57-10-3
**30.32**	**30.91**	**Methoxsalen**	**C_12_H_8_O_4_**	**298-81-7**
**31.22**	**6.39**	**α-Glyceryl linolenate**	**C_21_H_36_O_4_**	**18465-99-1**
**31.98**	**6.72**	**Isopimpinellin**	**C_13_H_10_O_5_**	**482-27-9**
32.76	0.64	3,3′,4,4′-Tetrahydrospirilloxanthin	C_42_H_64_O_2_	13833-01-7
**34.87**	**12.54**	**Triphenylphosphine oxide**	**C_18_H_15_OP**	**791-28-6**

Bold items indicate the major constituents identified by GC–MS in this plant sample.

**Table 2:** Antimicrobial activity of *Trodylium trachycarpum* plant extract solution (Tt1) and its synthesized copper nanoparticle form (Tt2) against different microbial strains.

Microorganism	Zone of Inhibition (mm)	
	Tt1	Tt2
*Escherichia coli*	NBG	3.0
*Pseudomonas aeruginosa*	2.0	2.7
*Klebsiella pneumoniae*	3.5	5.0
*Staphylococcus aureus*	1.0	3.0
*Staphylococcus epidermidis*	3.0	5.0
*Candida albicans*	2.0	3.4

Values represent the mean diameter of inhibition zones measured in millimeters (mm). NBG: no bacterial growth observed.

**Table 3:** Minimum Inhibitory Concentration of the Crude Methanol Extract (Tt1) versus Synthesized CuNPs (Tt2) against Microorganisms.

Types of Microorganism	Samples	Concentration (µg/mL)					Controls	
		125	250	500	1000	2000	+Ve	−Ve
*Escherichia coli*	Tt1	1.177 ± 0.07	0.972 ± 0.02	0.762 ± 0.08	0.400 ± 0.01	0.394 ± 0.03	0.833 ± 0.04	0.141 ± 0.02
	Tt2	0.840 ± 0.05	0.779 ± 0.05	0.126 ± 0.04	0.227 ± 0.04	0.332 ± 0.09		
*Pseudomonas aeruginosa*	Tt1	0.666 ± 0.09	0.419 ± 0.03	0.254 ± 0.01	0.205 ± 0.09	0.0165 ± 0.05	0.602 ± 0.01	0.119 ± 0.03
	Tt2	0.714 ± 0.10	0.377 ± 0.05	0.083 ± 0.04	0.221 ± 0.01	0.2985 ± 0.01		
*Klebsiella pneumoniae*	Tt1	1.009 ± 0.03	0.409 ± 0.04	0.774 ± 0.09	0.669 ± 0.09	0.604 ± 0.08	0.807 ± 0.04	0.096 ± 0.01
	Tt2	0.587 ± 0.11	0.027 ± 0.07	0.312 ± 0.05	0.088 ± 0.02	0.009 ± 0.01		
*Staphylococcus aureus*	Tt1	0.474 ± 0.10	0.475 ± 0.01	0.319 ± 0.06	0.099 ± 0.02	0.085 ± 0.05	0.575 ± 0.01	0.141 ± 0.06
	Tt2	0.911 ± 0.04	1.111 ± 0.13	0.297 ± 0.04	0.097± 0.03	0.258 ± 0.09		
*Staphylococcus epidermidis*	Tt1	0.605 ± 0.03	0.461 ± 0.08	0.478 ± 0.10	0.419± 0.07	0.379 ± 0.01	0.284 ± 0.02	0.099 ± 0.05
	Tt2	0.632 ± 0.11	0.419 ± 0.10	0.026 ± 0.07	0.153± 0.03	0.076 ± 0.01		
*Candida albicans*	Tt1	0.757 ± 0.08	0.838 ± 0.01	0.376 ± 0.06	0.340± 0.04	0.074 ± 0.05	0.699 ± 0.01	0.141 ± 0.04
	Tt2	0.378 ± 0.01	0.012 ± 0.05	0.032 ± 0.02	0.157± 0.07	0.216± 0.03		

(**Tt1**): methanol extract; (**Tt2**): synthesized copper nanoparticles. The underlined values are the MIC for each compound at a specified concentration that has been measured in optical density with a wavelength of 600 nm.

**Table 4:** Total phenolic and flavonoid contents of the methanolic extract (Tt1) and its synthesized copper nanoparticles (Tt2) derived from *Tordylium trachycarpum*.

Assay	Tt1 (Mean ± SD)	Tt2 (Mean ± SD)
Total phenol	26.22 ± 0.50 mg GAE/g	21.48 ± 0.61 mg GAE/g
Total flavonoids	46.90 ± 0.56 mg QE/g	39.03 ± 0.37 mg QE/g

Values are expressed as the mean ± standard deviation (SD) of three independent measurements (*n* = 3). GAE: gallic acid equivalents; QE: quercetin equivalents.

**Table 5:** Antioxidant activities of the methanolic extract (Tt1) and its synthesized copper nanoparticles (Tt2) derived from *Tordylium trachycarpum*.

Assay	Tt1 (Mean ± SD)	Tt2 (Mean ± SD)
DPPH (mg TE/g)	61.42 ± 1.30	88.42 ± 0.96
ABTS (mg TE/g)	135.18 ± 2.84	128.89 ± 4.96
FRAP (mg TE/g)	103.52 ± 8.16	113.15 ± 1.22
CUPRAC (mg TE/g)	63.04 ± 1.87	82.11 ± 0.72
PMA (mmol TE/g)	2.56 ± 0.12	1.38 ± 0.03
MCA (mg EDTA/g)	6.94 ± 0.69	11.42 ± 1.44

Values are expressed as the mean ± standard deviation (SD) of three independent measurements (*n* = 3)—TE: Trolox equivalents; EDTAE: ethylenediaminetetraacetic acid equivalents. Different letters within the same row (if applicable) indicate statistically significant differences (*p* < 0.05).

**Table 6:** Enzyme inhibitory activities of the methanolic extract (Tt1) and its copper nanoparticles (Tt2) derived from *Tordylium trachycarpum*.

Inhibition Assay	Samples (Mean ± SD)	
	Tt1	Tt2
AChE (mg GALAE/g)	2.29 ± 0.20	1.78 ± 0.09
BChE (mg GALAE/g)	0.89 ± 0.03	NA
Tyrosinase (mg KAE/g)	46.96 ± 3.18	52.62 ± 1.96
Amylase (mmol ACAE/g)	0.57 ± 0.01	0.35 ± 0.02
Glucosidase (mmol ACAE/g)	1.12 ± 0.02	NA

Values are expressed as the mean ± standard deviation (SD) of three independent measurements (*n* = 3). GALAE: galantamine equivalents; KAE: kojic acid equivalents; ACAE: acarbose equivalents. “NA” indicates undetectable or negligible inhibitory activity under the tested conditions.

## Data Availability

The original contributions presented in this study are included in the article. Further inquiries can be directed to the corresponding author. The plant specimen (*Tordylium trachycarpum* Boiss., family Apiaceae) used in this research is preserved as a voucher specimen (No. 7631) at the Herbarium of the Department of Biology, College of Science, Salahaddin University–Erbil, Iraq. All relevant taxonomic information is publicly available through the International Plant Names Index (IPNI) under LSID: urn:lsid:ipni.org:names:911555-1 (https://www.ipni.org/n/911555-1, accessed on 27 October 2025).
